# Association between Maternal Blood Glucose Levels during Pregnancy and Birth Outcomes: A Birth Cohort Study

**DOI:** 10.3390/ijerph20032102

**Published:** 2023-01-24

**Authors:** Doudou Zhao, Danmeng Liu, Wenhao Shi, Li Shan, Wentao Yue, Pengfei Qu, Chenghong Yin, Yang Mi

**Affiliations:** 1 Translational Medicine Center, Northwest Women’s and Children’s Hospital, No. 1616 Yanxiang Road, Xi’an 710061, China; 2 The Assisted Reproduction Center, Northwest Women’s and Children’s Hospital, No. 1616 Yanxiang Road, Xi’an 710061, China; 3 Department of Obstetrics and Gynecology, Northwest Women’s and Children’s Hospital, No. 1616 Yanxiang Road, Xi’an 710061, China; 4 Beijing Obstetrics and Gynecology Hospital, Capital Medical University, Beijing Maternal and Child Health Care Hospital, Beijing 100026, China

**Keywords:** blood glucose level, birth outcomes, fasting plasma glucose

## Abstract

Objective: This study aimed to investigate the relationship between maternal blood glucose levels during pregnancy and neonatal birth outcomes in Northwest China. Methods: This population-based cohort study included 10,010 first-trimester pregnant women who joined the birth cohort of the Northwest Women’s and Children’s Hospital from July 2018 to July 2020. Basic demographic characteristics, lifestyle and behavior patterns were collected. Oral glucose tolerance test (OGTT) results during the second trimester and pregnancy outcomes after childbirth were also collected. A generalized linear model was constructed to analyze the effects of blood glucose levels on neonatal birth outcomes. Results: We found that every 1 mmol/L increase in fasting plasma glucose (FPG) was associated with an increase in birth weight (*β* = 100.22 g, 95% confidence interval (*95%CI*): 81.91, 118.52), birth weight Z score (*β* = 0.23, *95%CI*: 0.19, 0.27) and birth weight Z centile (*β* = 6.72%, *95%CI*: 5.51, 7.94). Moreover, the risk of macrosomia, premature birth and being born large for gestational age (LGA) increased by 2.01 (*95%CI*: 1.67, 2.43), 1.35 (*95%CI*: 1.09, 1.66) and 1.80 (*95%CI*: 1.57, 2.07) times, respectively. Additionally, for every 1 mmol/L increase in FPG associated with a decrease in gestational age (*β* = −0.12 weeks, *95%CI*: −0.19, −0.06), the risk of SGA decreased by 0.70 (*OR* = 0.70, *95%CI*: 0.55, 0.89) times. Every 1 mmol/L increase in 1/2-h PG had similar outcomes as FPG, besides premature birth and SGA. Conclusions: Higher blood glucose in pregnant women may increase neonatal birth weight, decrease gestational age and lead to a higher risk of macrosomia, premature birth and LGA. Mothers should actively prevent and control hyperglycemia to promote maternal and infant health.

## 1. Introduction

Gestational diabetes mellitus (GDM) is the most common metabolic disorder during pregnancy and is defined as glucose intolerance that results in hyperglycemia initiated or first diagnosed during pregnancy. The prevalence of GDM differs widely depending on the diagnostic criteria used and population characteristics. The estimated global standardized prevalence of GDM was 14.0% in 2021, with the highest GDM prevalence in the Middle East and North Africa (27.6%), followed by Southeast Asia (20.8%), Western Pacific (14.7%) and Africa (14.2%) [1]. GDM affects one in seven live births globally, accounting for 85% of the 21.3 million global live births affected by diabetes during pregnancy [2]. In China, after the “two-child” and “three-child” policies were put into effect, the age of mothers increased rapidly. In parallel, the prevalence of GDM was 14.8% and has been growing over the past few decades [3]. Some studies have shown that hyperglycemia affects the health of the mother and birth outcomes, leads to maternal complications, increases the risk of macrosomia and LGA [4,5,6] and may lead to long-term deleterious effects [7,8,9].

Since the human fetus is highly dependent on glucose from the maternal circulation, glucose homeostasis transferred from the mother to the placenta is thought to be a major determinant of fetal development [10]. Substantial studies have shown that higher gestational blood glucose, whether in fasting or postprandial states in each trimester, is associated with an increased risk of adverse birth outcomes, even in nondiabetic pregnancies [11]. Indeed, higher random glucose levels in healthy pregnant women are associated with larger babies at birth and lower growth velocity in early childhood [12]. Hyperglycemia also contributes to childhood obesity, cardio metabolic disorders in women and growing long-term burden on future generations [13]. However, such studies are few in Northwest China, and are mostly based on small samples of hospital registry data and have not considered important confounders such as gestational weight gain, maternal lifestyle and environmental factors, which might compromise the reliability of conclusions to some extent.

Therefore, we analyzed 10,010 pregnant women who delivered at the Northwest Women’s and Children’s Hospital of Shaanxi Province from January 2018 to December 2020. We specifically assessed the relationship between maternal blood glucose levels during pregnancy and neonatal birth outcomes in Northwest China. The data presented herein provide insights on the early prevention of GDM, reducing the risk of maternal and neonatal complications caused by hyperglycemia, and reducing the medical burden to both countries and individuals.

## 2. Methods

### 2.1. Study Design and Population

Data were collected from a population-based prospective cohort study [14] on neonatal risk factors conducted in Xi’an, Shaanxi province, Northwest China, from January 2018 to December 2020. Pregnant women were initially recruited from the obstetrics outpatient clinic at the Northwest Women’s and Children’s Hospital, which is one of the largest obstetrics and gynecology hospitals in Northwest China. This study was approved by the Human Research Ethics Committee of the Beijing Obstetrics and Gynecology Hospital (no. 2018-KY-003-02) and Northwest Women’s and Children’s Hospital (no. 2018018). We obtained written informed consent from all participants, and the experimental procedure followed all ethical guidelines and regulations.

The following inclusion criteria were used to select patients for the study: (1) pregnant women who voluntarily joined the birth cohort and signed informed consent from January 2018 to December 2020; (2) 1 to 13^+6^ gestational weeks; (3) pregnancy care and delivery performed at Northwest Women’s and Children’s Hospital. The exclusion criteria were as follows: (1) under 20 years old; (2) presence of at least one of the following diseases before pregnancy: hypertension, GDM, hyperthyroidism, heart disease, chronic kidney disease or tuberculosis; (3) unable to answer questions accurately due to psychiatric symptoms or other serious illnesses. Termination or withdrawal criteria were as follows: (1) the subject developed serious diseases or died due to uncontrollable factors during the observation period; (2) the subject requested to be withdrawn or was lost to follow-up.

We excluded subjects with miscarriage or pregnancy induction (248 cases), twins (144 cases) and incomplete information (72 cases). This study only included pregnant women of singleton live births with well-defined pregnancy outcomes (10,010 cases).

### 2.2. Data Collection

This study was divided into three main stages. (1) Enrollment: After being recommended by doctors from the outpatient clinic, pregnant women voluntarily joined the birth cohort and completed the “Early Pregnancy Information Questionnaire” available at the EDC (Enterprise Data Center) cloud platform online system to collect general demographic characteristics and early pregnancy life behaviors. (2) Maternal medical examinations and complications during pregnancy were recorded during the second and third trimesters of pregnancy through the online EDC cloud platform and the hospital outpatient system. (3) Data on pregnancy outcome (date of delivery, birth weight, gestational age and other relevant information) were gathered through the hospital’s inpatient medical record system.

### 2.3. Study Variables

The birth weight Z score was calculated according to the INTERGROWTH-21st standard [15]: birth weight Z score = (neonatal birth weight − neonatal birth weight predicted median)/SD predicted value. Macrosomia was defined as a neonatal birth weight ≥ 4000 g. Low birth weight (LBW) was defined as a neonatal birth weight < 2500 g. Premature birth was defined as a baby born between 28 and 37 gestational weeks. Small for gestational age (SGA) refers to an infant whose birth weight is under the 10th percentile of the average weight for the same gestational age. Large for gestational age (LGA) refers to an infant whose birth weight is over the 90th percentile of the average weight for the same gestational age.

### 2.4. Blood Glucose Measurement

All pregnant women were subjected to a 75 g oral glucose tolerance test (OGTT) at 24 - 28 weeks of gestation. Abnormal blood glucose levels were diagnosed according to American Diabetes Association criteria: fasting plasma glucose (FPG) ≥ 5.1 mmol/L, 1-h plasma glucose (PG) ≥ 10.0 mmol/L or 2-h PG ≥ 8.5 mmol/L.

### 2.5. Covariates

Considering data from previous studies [16,17], we collected confounding variables related to neonatal weight, such as maternal demographic characteristics and lifestyles. Confounding variables included maternal age (< 35, ≥ 35), ethnicity (Han, other), education (senior high school or lower, junior or regular college, graduate or above), yearly average household income (low: < 50,000 Chinese yuan (CNY), medium: 50,000 ~ 200,000 CNY, high: > 200,000 CNY), body mass index (BMI) before pregnancy (underweight: < 18.5 kg/m^2^, normal: 18.5 ~ 23.9 kg/m^2^, overweight: 24.0 ~ 27.9 kg/m^2^, obesity: ≥ 28.0 kg/m^2^), gestational weight gain (GWG) (suitable, appropriate, excessive), first pregnancy (yes, no), complications in previous pregnancies (yes, no), fetal sex (male, female), smoking or passive smoking (yes, no), fever or cold (yes, no) and the use of multidimensional nutrients or folic acid supplements (yes, no). “Pregnancy period” refers to the period from 3 months before pregnancy to the end of pregnancy. “Smoking or passive smoking” refers to smoking at least one cigarette or passive inhalation of smoke for more than 15 min at least 1 day per week during pregnancy. “Maternal fever or cold” refers to having a fever or cold at least 1 time during pregnancy. “Supplementation of folic acid or multidimensional nutrients” refers to mothers taking folic acid or multidimensional nutrients for more than 30 days during pregnancy. “Complications in previous pregnancy” refers to mothers who developed gestational hypertension, GDM or thyroid disease during a previous pregnancy. BMI was obtained by dividing the mother’s pre-pregnancy weight by the square of their height (kg/m^2^). GWG was classified as insufficient, appropriate or excessive based on pre-gestational BMI according to 2009 Institute of Medicine (IOM) criteria. Specifically, for underweight women, appropriate GWG ranges from 12.5 to 18 kg; for normal women, 11.5 to 15.9 kg; for overweight women, 7 to 11.5 kg; and for obese women, 5 to 9 kg.

### 2.6. Statistical Analysis

Categorical variables are expressed as frequency and percentages, while continuous variables are expressed as mean and standard deviation (SD). In univariate comparisons, categorical variables were compared between groups using the χ^2^ test. A t-test or analysis of variance (ANOVA) was used to compare values between groups for continuous variables. We constructed a generalized linear model (GLM) to explore the association between blood glucose levels during pregnancy and neonatal birth outcomes. Continuous outcome variables, such as neonatal gestational age, selected the connection function as identity: *g*^−1^(*μ*) = *μ*. Binary outcome variables, such as low birth weight, selected the connection function as logit: *η* = l*n* (*μ*/1 − *μ*), where *μ* is the conditional probability of the offspring outcome variable. Possible confounding variables were included in the multivariate analysis. We drew the trend for macrosomia risk and neonatal birth weight, and gestational age despite elevated blood glucose levels by restricted cubic spline and smoothing spline, respectively. Moreover, we used the GLM to evaluate the effects of changes in blood glucose levels on neonatal birth outcomes. As shown in Supplementary Table S1, there was no high autocorrelation between covariates through correlation analysis (|r| < 0.7). All statistical analyses were performed in SAS version 9.4 (SAS Institute, Cary, NC, USA). *p* < 0.05 indicated a significant difference.

## 3. Results

### 3.1. Participant Characteristics

Table 1 compares maternal characteristics in hyperglycemic women with those of women with normal blood glucose levels. Mothers in the high blood glucose group were more likely to be older, overweight or obese, have lower educational and economical levels, have insufficient GWG, have had a previous pregnancy, have greater incidence of complications from previous pregnancy and have higher blood glucose levels during pregnancy compared to the normal blood glucose group (*p* < 0.05).

### 3.2. High Blood Glucose and Birth Outcomes

As shown in Table 2, high blood glucose could increase the birth weight (*β* = 59.21 g, *95%CI*: 40.86, 77.56), birth weight Z score (*β* = 0.12, *95%CI*: 0.08, 0.17), birth weight Z centile (*β* = 3.83%, *95%CI*: 2.62, 5.05), decrease the gestational age (*β* = −0.09 w, *95%CI*: −0.15, −0.02) and lead to a higher risk of macrosomia (*OR* = 1.55, *95%CI*: 1.27, 1.90) and LGA (*OR* = 1.36, *95%CI*: 1.19, 1.59) in the adjusted model. Supplementary Table S2 presents the outcomes of subgroup analyses, in which all effects of subgroups were positive (*β > 0 or OR*  >  1). The association between maternal high blood glucose and birth weight and macrosomia was relatively stable, although some effects were not statistically significant.

### 3.3. Blood Glucose Levels and Birth Outcomes

**Figure 1:**
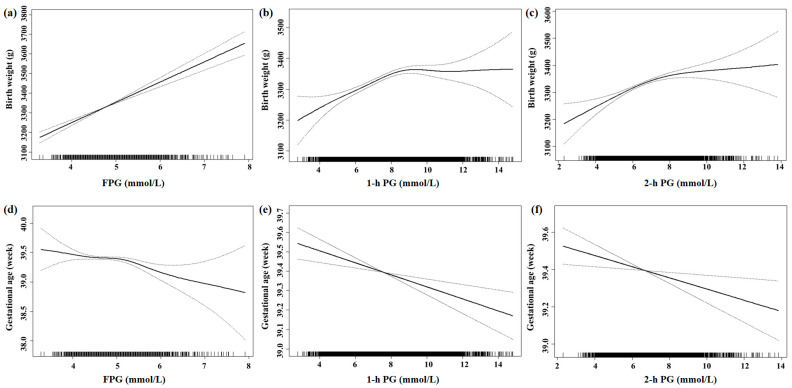
Association of blood glucose levels with birth weight and gestational age by smoothing spline. With the increase of FPG (**a**), the birth weight showed a linear upward trend. With the increase of 1-h PG (**b**) and 2-h PG (**c**), the birth weight showed a non-linear upward trend. With the increase of FPG (**d**), the gestational age showed a non-linear downward trend. With the increase of 1-h PG (**e**) and 2-h PG (**f**), the gestational age showed a linear downward trend.

**Figure 2:**
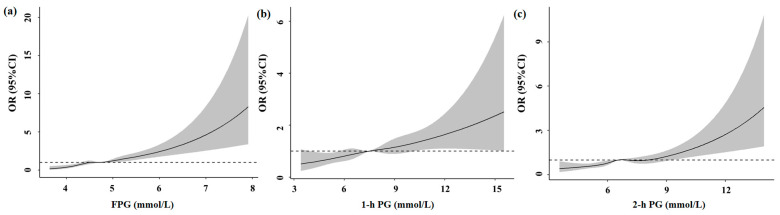
Association of blood glucose levels with macrosomia by restricted cubic spline. With the increase of FPG (**a**), 1-h PG (**b**) and 2-h PG (**c**), the odds ratios of macrosomia showed a non-linear upward trend.

After adjustments, every 1 mmol/L increase in FPG could increase birth weight (*β* = 100.22 g, *95%CI*: 81.91, 118.52), birth weight Z score (*β* = 0.23, *95%CI*: 0.19, 0.27) and birth weight Z centile (*β* = 6.72%, *95%CI*: 5.51, 7.94), and increase the risk of macrosomia, premature birth and LGA by 2.01 (*OR* = 2.01, *95%CI*: 1.67, 2.43), 1.35 (*OR* = 1.35, *95%CI*: 1.09, 1.66) and 1.80 (*OR* = 1.80, *95%CI*: 1.57, 2.07) times, respectively. Moreover, every 1 mmol/L increase in FPG decreased gestational age (*β* = −0.12 w, *95%CI*: −0.19, −0.06) and decreased the risk of SGA by 0.70 (*OR* = 0.70, *95%CI*: 0.55, 0.89) times. Every 1 mmol/L increase in 1-h PG and 2-h PG had similar outcomes as FPG, besides premature birth and SGA (Table 3). Supplementary Table S3 presents the associations of abnormal blood glucose and birth outcomes; the effects of FPG and 1-h PG on birth weight were statistically significant. Every 1 mmol/L increase in FPG (*β* = 1.83 kg, *95%CI*: 0.56, 3.10) and 1-h PG (*β* = 0.42 kg, *95%CI*: 0.09, 0.75) could increase maternal weight before delivery (Supplementary Table S4). As shown in Figure 1 and Figure 2, maternal hyperglycemia was associated with increased neonatal birth weight and higher risk of macrosomia and a decreased gestational age. Supplementary Figure S1 shows that FPG was linearly associated with increased neonatal birth weight, similar to Figure 1 after including the squared FPG term in the model. The effect of FPG was the most significant among the three blood glucose values used, followed by 2-h PG.

### 3.4. Blood Glucose Changes and Birth Outcomes

As shown in Table 4 and Table 5, for every 1 mmol/L change in blood glucose between fasting and 1-h PG, the birth weight, birth weight Z score and birth weight Z centile increased by 12.71 g, 0.03 and 0.79%, respectively. In parallel, the gestational age decreased by 0.03 weeks, and the risk of macrosomia and LGA increased by 1.09 and 1.08 times, respectively. In addition, a 1 mmol/L change in glucose levels from 1-h to 2-h PG was associated with increased birth weight (+6.19 g), birth weight Z centile (+0.37%) and risk of premature birth (+1.07 times). From fasting to 2-h later, for every 1 mmol/L change in blood glucose, the birth weight, birth weight Z score and birth weight Z centile increased by 13.01 g, 0.03 and 0.83%, while the risk of macrosomia and LGA increased by 1.13 and 1.10 times, respectively.

In addition, 1% changes in blood glucose ratio from fasting to 1-h increased the birth weight, birth weight Z score and birth weight Z centile by 39.95 g, 0.08 and 2.39%, respectively. The gestational age decreased by 0.1 weeks, while the risk of LGA increased by 1.26 times. Changes in the ratio between fasting and 2-h PG were associated with similar findings.

This study explored the relationship between maternal blood glucose during pregnancy and neonatal birth outcomes in Northwest China. Mothers with every 1 mmol/L increase in FPG and 1/2-h PG were associated with the following birth outcomes: increased birth weight, birth weight Z score, birth weight Z centile, decreased gestational age and higher risk of macrosomia and LGA. Meanwhile, every 1 mmol/L increase in FPG was also associated with higher risk of premature birth and lower risk of SGA. Rises in blood glucose levels were associated with increased birth weight and risk of macrosomia, as well as decreased gestational age. Finally, changes in the ratio of maternal blood glucose levels from fasting to 2-h PG were associated with higher neonatal weight and shorter gestational age in different degrees.

This study found that mothers with high blood glucose during pregnancy had babies with increased birth weight and risk of macrosomia and LGA, which is consistent with conclusions of previous studies. Indeed, a prospective cohort study conducted in Zhou Shan (Zhejiang Province) found that abnormal blood glucose was positively correlated with neonatal birth weight (*β* = 99.5, *p* < 0.001), weight Z score (*β* = 0.23, *p* < 0.001), macrosomia (*OR* = 2.13, *95%CI*: 1.34, 3.40) and LGA (*OR* = 1.79, *95%CI*: 1.11, 2.89) [18]. Maternal hyperglycemia led to a higher risk of macrosomia [19,20], the need for blue light therapy and higher umbilical cord blood C-peptide and insulin concentrations compared to babies born from normal glycemic mothers. Moreover, the mean birth weight in the abnormal blood glucose group was significantly higher (*p* = 0.004) [21]. A retrospective study found that the incidence of cesarean section, premature birth, macrosomia, neonatal respiratory distress and neonatal hypoglycemia was associated with GDM during the first trimester [22].

Another cohort study continuously measured maternal glucose levels and found that a 1-unit increase in maternal mean nighttime glycemia was associated with a 6.0 (*95%CI*: 0.4, 11.5) percentage-point increase in birth weight percentile [23]. Meanwhile, a prospective study conducted in Guangdong found that for every 1 mmol/L increase in FPG, the risk of LGA and macrosomia increased by 2.01 (*95%CI*: 1.54, 5.88) and 2.74 (*95%CI*: 1.85, 7.60), respectively [24]. A regression discontinuity analysis in England [25] found for each 1 mmol/L increase in FPG that the observed increase in birth weight Z score increased by 0.48 (*95%CI*: 0.39, 0.57) and the odds ratio of LGA increased by 2.61 times (*95%CI*: 1.86, 3.66). Higher early FPG levels were associated with restricted fetal growth in the second trimester, followed by a compensatory growth starting in the third trimester that culminated in a significant weight gain and an increased risk of LGA [26]. A cohort of offspring exposed to hyperglycemia first detected during pregnancy found that maternal postprandial 2-h PG in the third trimester of pregnancy was significantly associated with weight Z scores at birth and at preschool age, SGA and LGA at birth [27]. Indeed, OGTT values may help to identify varying degrees of maternal and fetal risk, since GDM mothers that experience two or more abnormal PGs have a more severe disruption of glucose homeostasis and insulin sensitivity compared to GDM mothers who experienced only one altered PG [28]. In this study, elevated blood glucoses during pregnancy increased the birth weight and led to a higher risk of macrosomia and LGA, but the statistical association was only found between higher FPG and SGA. This suggests that one should closely monitor FPG.

Furthermore, our study examined how ratio changes from fasting to 1/2-h PG were associated with birth outcomes. Interestingly, a previous study highlighted that 2-h post-load serum glucose level was an independent predictor of neonatal birth weight in healthy nondiabetic pregnancies [29]. Another study found that women with abnormal FPG, regardless of it being at 1-h or 2-h PG, have an increased risk of LGA births [30]. In general, associations found for FPG are stronger than those found for post-load blood glucose concentration [11]. Here, we found that ratio changes from fasting to 1/2-h PG were more significantly associated with neonatal weight, perhaps because these changes were based on FPG and FPG is more closely associated with neonatal weight and had a better predictive effect.

Some studies have found that maternal hyperglycemia during pregnancy increase the risk of preterm birth [31,32]. Specifically, a study conducted in South Africa found that, compared with the control group, the incidence of cesarean section, premature birth, neonatal respiratory distress and neonatal hypoglycemia in women with high blood glucose during pregnancy was significantly higher [22]. Feng and colleagues found that elevated FPG was a key risk factor for preterm birth (*OR* = 1.46, *p* < 0.001), stillbirth (*OR* = 1.43, *p* < 0.001) and neonatal respiratory distress syndrome (*OR* = 1.32, *p* < 0.001) [33]. Indeed, another study found that pregnant women with an abnormal FPG have a 1.42-fold increased risk of spontaneous preterm birth (*95%CI*: 1.15, 1.77) [34]. Here, we found that maternal hyperglycemia during pregnancy could decrease the gestational age, but we only found associations between elevated FPG and preterm birth in the multivariate analysis. This might be due to the low incidence of preterm birth in our study, which needs to be corroborated by larger, multi-center studies.

Overall, the effect values (*OR*, *β*) of blood glucose levels on birth outcomes in this study seem to be lower than in developed countries and economically developed areas such as the coastal provinces in China. It is well known that regional variations in the prevalence of GDM, due to diagnostic criteria, ethnicity, lifestyle and environmental factors [35], and influence its effect on birth outcomes. Underlying biological mechanisms of how hyperglycemia affects embryonic development include oxidative stress, epigenetic modifications and mitochondrial function [36,37,38,39,40,41]. The rate of glucose delivery to the fetus is controlled by a maternal–fetal glucose concentration gradient across the placenta [42]. Hyperglycemia-induced apoptosis of non-proliferating syncytial trophoblast cells leaves incomplete pores in the placenta, which results in a large influx of glucose into the fetal circulation [43]. Thus, an increased amount of glucose enters the fetus through the placenta, stimulating fetal pancreatic islet B cells to secrete insulin under stress, which increases the fetal metabolic rate and the synthesis of protein and fat. Ultimately, this leads to increased fetus growth and birth weight [44]. In parallel, stimulation of fetal insulin secretion decreases fetal glycaemia, which increases the maternal–fetal glucose gradient, leading to rapid fetal growth and excess fat deposition [45].

Some studies have shown that increased protein in poultry, fish, nuts and soy foods in the diet of pregnant women may reduce the risk of GDM [46,47,48]. In addition, physical activity helps patients achieve adequate glycemic control and reduce their insulin use [49]. One study found that plasma microRNAs-16-5p, -17-5p and -20a-5p are potential biomarkers for the diagnosis of GDM [50]. Therefore, improved health care and regular monitoring of blood glucose levels should be strengthened during pregnancy to reduce the incidence and damage of hyperglycemia [51].

This was a large, population-based, prospective study that investigated the impact of maternal hyperglycemia during pregnancy on birth outcomes and provided high-quality data in an underexplored population using statistics. We introduced indicators that comprehensively reflect neonatal developmental status, such as birth weight Z score, birth weight Z centile and blood glucose changes to comprehensively analyze the influence of blood glucose levels on birth outcomes. Overall, the data contained in this study might enlighten policy makers and help guide strategies to prevent hyperglycemia in pregnant women. However, some limitations should be acknowledged. First, the research subjects were all from Northwest Women’s and Children’s Hospital, which might induce a selection bias. Second, some confounding factors in this study, such as cold or fever before pregnancy and early pregnancy, were obtained through recall, and we cannot exclude a recall bias. Third, we did not obtain maternal ambulatory information on gestational blood glucose levels and glycosylated hemoglobin, which might influence the effect of blood glucose on neonatal birth outcomes.

## 4. Conclusions

In conclusion, higher blood glucose during pregnancy has adverse effects on both the mother and the newborn. This study found that every 1 mmol/L increase in maternal PG levels during pregnancy was associated with increased birth weight, decreased gestational age and higher risk of macrosomia and LGA. Additionally, elevated FPG was also associated with a higher risk of premature birth and a lower risk of SGA. Health education and pregnancy care for women of childbearing age should be strengthened, and corresponding measures should be actively taken to prevent and treat hyperglycemia to promote prenatal and postnatal care.

## Figures and Tables

**Table 1:** General characteristics of study subjects (*n* = 10,010).

Characteristics	High Blood Glucose ^a^ *n* (%) (*n* = 2464)	Normal Blood Glucose *n* (%) (*n* = 7546)	*χ^2^/t*	*p* Value
Age				
<35	2147 (23.45)	7008 (76.55)	77.223	<0.001
≥35	317 (37.08)	538 (62.92)		
Ethnicity				
Han	2430 (24.66)	7424 (75.34)	0.679	0.410
Other	34 (21.79)	122 (78.21)		
Education				
Senior high school or lower	346 (28.11)	885 (71.89)	10.701	0.005
Junior or regular college	1763 (24.38)	5467 (75.62)		
Graduate or above	355 (22.92)	1194 (77.08)		
BMI before pregnancy (kg/m^2^)				
Underweight (<18.5)	236 (14.97)	1341 (85.03)	287.968	<0.001
Normal (18.5~23.9)	1577 (23.21)	5217 (76.79)		
Overweight (24.0~27.9)	532 (39.55)	813 (60.45)		
Obesity (≥28.0)	119 (40.48)	175 (59.52)		
Household income				
Low	324 (27.76)	843 (72.24)	9.167	0.010
Medium	1644 (24.58)	5045 (75.42)		
High	496 (23.03)	1658 (76.97)		
Fetal sex				
Male	1279 (24.84)	3869 (75.16)	0.300	0.584
Female	1185 (24.37)	3677 (75.63)		
Gestational weight gain/kg				
Appropriate	1021 (21.90)	3642 (78.10)	124.775	<0.001
Insufficient	755 (33.54)	1496 (66.46)		
Excessive	688 (22.22)	2408 (77.78)		
First pregnancy				
No	1201 (27.23)	3210 (72.77)	28.995	<0.001
Yes	1263 (22.56)	4336 (77.44)		
Complications from previous pregnancy				
No	2288 (23.97)	7256 (76.03)	45.566	<0.001
Yes	176 (37.77)	290 (62.23)		
Smoke or passive smoke				
No	2057 (24.29)	6412 (75.71)	3.166	0.075
Yes	407 (26.41)	1134 (73.59)		
Folic acid or multidimensional nutrients supplement				
No	42 (26.75)	115 (73.25)	0.392	0.531
Yes	2422 (24.58)	7431 (75.42)		
Fever or cold				
No	1938 (24.63)	5931 (75.37)	0.003	0.954
Yes	526 (24.57)	1615 (75.43)		
FPG (mmol/L)	5.26 ± 0.52	4.61 ± 0.27	59.082	<0.001
1-h PG (mmol/L)	9.07 ± 1.88	7.08 ± 1.33	48.790	<0.001
2-h PG (mmol/L)	7.76 ± 1.57	6.32 ± 0.95	42.992	<0.001

^a^ Defined according to American Diabetes Association criteria.

**Table 2:** Association of high blood glucose and birth outcomes (*n* = 10,010).

	High Blood Glucose *n* (%) (*n* = 2464)	Normal Blood Glucose *n* (%) (*n* = 7546)	Univariate Analysis	Multivariate Analysis ^c^
			*β*/*OR* (95%*CI*), *p*	Adjusted *β*/*OR* (95%*CI*), *p*
Birth weight				
Birth weight/g (*Mean* ± *SD*)	3349.54 ± 459.20	3322.27 ± 457.55	27.27 (6.45, 48.09), 0.010	59.21 (40.86, 77.56), <0.001 ^a^
Birth weight Z score (*Mean* ± *SD*)	0.33 ± 0.92	0.19 ± 0.91	0.14 (0.10, 0.18), <0.001	0.12 (0.08, 0.17), <0.001 ^b^
Birth weight Z centile (*Mean* ± *SD*)	59.57 ± 26.87	55.45 ± 26.45	4.12 (2.92, 5.33), <0.001	3.83 (2.62, 5.05), <0.001 ^b^
LBW	67 (2.72)	192 (2.54)	1.07 (0.81, 1.42), 0.635	0.90 (0.62, 1.30), 0.580 ^a^
Macrosomia	170 (6.90)	414 (5.49)	1.28 (1.06, 1.54), 0.010	1.55 (1.27, 1.90), <0.001 ^a^
Gestational age				
Gestational age/week (Mean ± SD)	39.22 ± 1.32	39.45 ± 1.44	−0.23 (−0.30, −0.17), <0.001	−0.09 (−0.15, −0.02), 0.008 ^b^
Premature birth	127 (5.15)	276 (3.66)	1.43 (1.15, 1.78), 0.001	1.10 (0.88, 1.38), 0.398 ^b^
SGA and LGA				
SGA	107 (4.34)	353 (4.68)	0.93 (0.74, 1.15), 0.490	0.97 (0.77, 1.22), 0.799 ^b^
LGA	338 (13.72)	768 (10.18)	1.40 (1.22, 1.61), <0.001	1.36 (1.19, 1.59), <0.001 ^b^

^a^ Adjusted for maternal age, education, household income, BMI before pregnancy, gestational weight gain, first pregnancy, complications from previous pregnancy, insulin injection and gestational week. ^b^ Adjusted for maternal age, education, household income, BMI before pregnancy, gestational weight gain, first pregnancy, complications from previous pregnancy and insulin injection. ^c^ After evaluating Pearson Chi-Square and C statistics, multivariate regression models showed an appropriate goodness of fit.

**Table 3:** Multivariate analysis of blood glucose levels during pregnancy and birth outcomes.

Blood Glucose During Pregnancy (Continuous Variable)	FPG ^c^		1-h PG ^c^		2-h PG ^c^	
	*Adjusted* *β/OR* (*95%CI*)	*p*	*Adjusted* *β/OR (95%CI)*	*p*	*Adjusted* *β/OR (95%CI)*	*p*
Birth weight						
Birth weight/g ^a^	100.22 (81.91, 118.52)	<0.001	17.51 (12.82, 22.20)	<0.001	22.66 (16.46, 28.86)	<0.001
Birth weight Z score ^b^	0.23 (0.19, 0.27)	<0.001	0.04 (0.03, 0.05)	<0.001	0.05 (0.04, 0.06)	<0.001
Birth weight Z centile ^b^	6.72 (5.51, 7.94)	<0.001	1.12 (0.81, 1.43)	<0.001	1.48 (1.07, 1.90)	<0.001
LBW ^a^	0.77 (0.53, 1.12)	0.168	0.99 (0.90, 1.09)	0.829	0.95 (0.84, 1.08)	0.421
Macrosomia ^a^	2.01 (1.67, 2.43)	<0.001	1.13 (1.07, 1.19)	<0.001	1.21 (1.13, 1.29)	<0.001
Gestational age						
Gestational age/week ^b^	−0.12 (−0.19, −0.06)	<0.001	−0.03 (−0.05, −0.02)	<0.001	−0.03 (−0.05, −0.01)	0.004
Premature birth ^b^	1.35 (1.09, 1.66)	0.005	1.05 (0.99, 1.12)	0.077	1.01 (0.93, 1.09)	0.869
SGA and LGA						
SGA ^b^	0.70 (0.55, 0.89)	0.004	0.98 (0.92, 1.04)	0.458	0.94 (0.87, 1.02)	0.145
LGA ^b^	1.80 (1.57, 2.07)	<0.001	1.12 (1.07, 1.16)	<0.001	1.16 (1.11, 1.22)	<0.001

^a^ Adjusted for maternal age, education, household income, BMI before pregnancy, gestational weight gain, first pregnancy, complications from previous pregnancy, insulin injection and gestational week. ^b^ Adjusted for maternal age, education, household income, BMI before pregnancy, gestational weight gain, first pregnancy, complications from previous pregnancy and insulin injection. ^c^ After evaluating Pearson Chi-Square and C statistics, multivariate regression models showed an appropriate goodness of fit.

**Table 4:** Multivariate analysis of blood glucose changes during pregnancy and birth outcomes.

	1-h PG-FPG ^c^		2-h PG-1-h PG ^c^		2-h PG-FPG ^c^	
	*Adjusted* *β/OR* (*95%CI*)	*p*	*Adjusted* *β/OR* (*95%CI*)	*p*	*Adjusted* *β/OR* (*95%CI*)	*p*
Birth weight						
Birth weight/g ^a^	12.71 (7.66, 17.77)	<0.001	6.19 (0.71, 11.67)	0.027	13.01 (6.32, 19.70)	<0.001
Birth weight Z score ^b^	0.03 (0.02, 0.04)	<0.001	0.01 (−0.01, 0.02)	0.050	0.03 (0.01, 0.04)	<0.001
Birth weight Z centile ^b^	0.79 (0.45, 1.12)	<0.001	0.37 (0.01, 0.73)	0.047	0.83 (0.39, 1.27)	<0.001
LBW ^a^	1.01 (0.91, 1.12)	0.865	1.03 (0.92, 1.14)	0.649	0.98 (0.85, 1.12)	0.734
Macrosomia ^a^	1.09 (1.02, 1.15)	0.006	1.02 (0.95, 1.08)	0.621	1.13 (1.05, 1.22)	0.002
Gestational age						
Gestational age/week ^b^	−0.03 (−0.05, −0.01)	0.002	−0.02 (−0.04, 0.01)	0.059	−0.02 (−0.04, 0.01)	0.078
Premature birth ^b^	1.04 (0.97, 1.11)	0.253	1.07 (1.01, 1.15)	0.048	0.96 (0.89, 1.05)	0.396
SGA and LGA						
SGA ^b^	1.00 (0.94, 1.06)	0.973	1.01 (0.95, 1.08)	0.680	0.98 (0.90, 1.06)	0.584
LGA ^b^	1.08 (1.04, 1.13)	<0.001	1.03 (0.98, 1.08)	0.206	1.10 (1.04, 1.16)	0.001

^a^ Adjusted for maternal age, education, household income, BMI before pregnancy, gestational weight gain, first pregnancy, complications from previous pregnancy, insulin injection and gestational week. ^b^ Adjusted for maternal age, education, household income, BMI before pregnancy, gestational weight gain, first pregnancy, complications from previous pregnancy and insulin injection. ^c^ After evaluating Pearson Chi-Square and C statistics, multivariate regression models showed an appropriate goodness of fit.

**Table 5:** Multivariate analysis of blood glucose change ratios during pregnancy and birth outcomes.

	(1-h PG-FPG)/FPG ^c^		(2-h PG-1-h PG)/1-h PG ^c^		(2-h PG-FPG)/FPG ^c^	
	*Adjusted* *β/OR* (*95%CI*)	*p*	*Adjusted* *β/OR* (*95%CI*)	*p*	*Adjusted* *β/OR* (*95%CI*)	*p*
Birth weight						
Birth weight/g ^a^	39.95 (15.67, 64.23)	0.001	34.97 (−6.44, 76.37)	0.098	37.50 (5.77, 69.22)	0.021
Birth weight Z score ^b^	0.08 (0.03, 0.14)	0.004	0.07 (−0.02, 0.16)	0.143	0.08 (0.01, 0.15)	0.030
Birth weight Z centile ^b^	2.39 (0.78, 4.00)	0.004	1.96 (−0.79, 4.71)	0.162	2.35 (0.24, 4.45)	0.029
LBW ^a^	1.04 (0.63, 1.71)	0.873	1.04 (0.44, 2.45)	0.930	0.92 (0.48, 1.78)	0.814
Macrosomia ^a^	1.24 (0.94, 1.64)	0.128	1.07 (0.67, 1.71)	0.765	1.41 (0.98, 2.04)	0.066
Gestational age						
Gestational age/week ^b^	−0.10 (−0.19, −0.02)	0.018	−0.09 (−0.24, 0.05)	0.221	−0.07 (−0.19, 0.04)	0.190
Premature birth ^b^	1.11 (0.81, 1.52)	0.500	1.73 (0.98, 3.07)	0.060	0.78 (0.52, 1.18)	0.240
SGA and LGA						
SGA ^b^	1.04 (0.77, 1.39)	0.814	1.12 (0.67, 1.86)	0.665	0.95 (0.64, 1.40)	0.787
LGA ^b^	1.26 (1.03, 1.55)	0.025	1.24 (0.87, 1.75)	0.234	1.31 (1.01, 1.71)	0.049

^a^ Adjusted for maternal age, education, household income, BMI before pregnancy, gestational weight gain, first pregnancy, complications from previous pregnancy, insulin injection and gestational week. ^b^ Adjusted for maternal age, education, household income, BMI before pregnancy, gestational weight gain, first pregnancy, complications from previous pregnancy and insulin injection. ^c^ After evaluating Pearson Chi-Square and C statistics, multivariate regression models showed an appropriate goodness of fit.

## Data Availability

The data presented in this study are available on request from the corresponding author. The data are not publicly available due to project regulations.

## Supplementary Materials

The following supporting information can be downloaded at: https://www.mdpi.com/article/10.3390/ijerph20032102/s1. Table S1. The autocorrelations between covariates. Table S2. Association between pregnancy hyperglycemia and birth weight and macrosomia in different subgroups. Table S3. Multivariate analysis of abnormal blood glucose levels during pregnancy and birth outcomes. Table S4. Association of blood glucose levels and maternal weight before delivery (N = 10,010). Supplementary Figure S1. Association of FPG with birth weight by smoothing spline. Adjusted for: squared FPG.
