# Supplementary material for: Association between Maternal Blood Glucose Levels during Pregnancy and Birth Outcomes: A Birth Cohort Study
Source: Int J Environ Res Public Health. 2023 Jan 24;20(3):2102. doi: 10.3390/ijerph20032102 (PMC9915873; doi:10.3390/ijerph20032102)
Supplement: Supplementary file 1 [file ijerph-20-02102-s001.zip › ijerph-2042482-supplementary.pdf]

Supplementary table S1. The autocorrelations between covariates.

r	Age	Education	BMI	Household income	First pregnancy	Complications	GWG	Insulin injection
Age	1.0000	0.0006	0.0108	0.0332	-0.2457	0.0614	0.0136	0.0061
Education	0.0006	1.0000	-0.0910	0.2689	0.1144	-0.0188	-0.0744	0.0061
BMI	0.0108	-0.0910	1.0000	-0.0555	-0.0143	0.0354	0.1808	0.0345
Household income	0.0332	0.2689	-0.0555	1.0000	-0.0454	0.0243	-0.0316	-0.0043
First pregnancy	-0.2457	0.1144	-0.0143	-0.0454	1.0000	-0.2490	-0.0257	-0.0054
Complications	0.0614	-0.0188	0.0354	0.0243	-0.2490	1.0000	0.0158	0.0190
GWG	0.0136	-0.0744	0.1808	-0.0316	-0.0257	0.0158	1.0000	-0.0010
Insulin injection	0.0061	0.0061	0.0345	-0.0043	-0.0054	0.0190	-0.0010	1.0000

Supplementary table S2. Association between pregnancy hyperglycemia and birth weight and macrosomia in different subgroups.

	Birth weight		Macrosomia	
	Adjusted β (95%CI) [a]	*P*	*Adjusted OR (95%CI)* [a]	*P*
Age				
< 35	65.62 (46.26, 84.98)	<0.001	1.58 (1.28, 1.94)	<0.001
≥ 35	17.85 (-37.23, 72.94)	0.525	1.39 (0.72, 2.71)	0.324
Education				
Senior high school or lower	62.93 (10.50, 115.36)	0.019	1.51 (0.89, 2.56)	0.128
Junior or regular college	69.11 (47.66, 90.55)	<0.001	1.56 (1.24, 1.97)	<0.001
Graduate or above	4.91 (-41.91, 51.73)	0.837	1.59 (0.89, 2.85)	0.120
BMI before pregnancy (kg/m^2)				
Underweight (< 18.5)	13.80 (-41.06, 68.66)	0.622	1.25 (0.57, 2.76)	0.571
Normal (18.5 ~ 23.9)	51.13 (29.37, 72.89)	<0.001	1.51 (1.17, 1.96)	0.002
Overweight (24.0 ~ 27.9)	110.50 (64.80, 156.19)	<0.001	1.88 (1.26, 2.80)	0.002
Obesity (≥ 28.0)	94.24 (-19.35, 207.83)	0.104	1.06 (0.48, 2.33)	0.881
Household income				

Low	93.59 (42.08, 145.09)	<0.001	2.02 (1.17, 3.48)	0.012
Medium	62.89 (40.73, 85.04)	<0.001	1.52 (1.19, 1.93)	<0.001
High	31.59 (-9.71, 72.88)	0.134	1.42 (0.89, 2.25)	0.140
Gestational weight gain /kg				
Appropriate	72.65 (45.99, 99.31)	<0.001	1.72 (1.26, 2.34)	<0.001
Insufficient	3.86 (-29.86, 37.58)	0.823	1.08 (0.63, 1.94)	0.792
Excessive	95.44 (58.98, 131.89)	<0.001	1.55 (1.16, 2.06)	0.003
First pregnancy				
No	56.03 (28.91, 83.14)	<0.001	1.48 (1.21, 1.96)	0.006
Yes	64.35 (39.62, 89.09)	<0.001	1.62 (1.22, 2.06)	<0.001

[a] Adjusted for: maternal age, education, household income, BMI before pregnancy, gestational weight gain, first pregnancy, complications from previous pregnancy, insulin injection and gestational week, respectively.

Supplementary table S3. Multivariate analysis of abnormal blood glucose levels during pregnancy and birth outcomes.

Blood glucose during pregnancy (normal *vs* abnormal)	FPG[c]		1-h PG[c]		2-h PG[c]	
	Adjusted β/OR (95%CI)	*P*	*Adjusted β/OR (95%CI)*	*P*	*Adjusted β/OR (95%CI)*	*P*
Birth weight						
Birth weight/g [a]	76.64 (56.32, 96.96)	<0.001	21.87 (-7.23, 50.97)	0.141	32.79 (3.25, 62.33)	0.030
Birth weight Z score [b]	0.17 (0.12, 0.21)	<0.001	0.04 (-0.02, 0.11)	0.189	0.07 (-0.01, 0.13)	0.055
Birth weight Z centile [b]	5.02 (3.67, 6.37)	<0.001	1.61 (-0.32, 3.54)	0.101	1.80 (-0.16, 3.76)	0.072
LBW [a]	1.00 (0.67, 1.49)	0.985	1.27 (0.76, 2.12)	0.357	0.74 (0.41, 1.35)	0.332
Macrosomia [a]	1.66 (1.34, 2.04)	<0.001	1.35 (0.97, 1.86)	0.072	1.61 (1.17, 2.21)	0.003
Gestational age						
Gestational age/week [b]	-0.08 (-0.15, -0.01)	0.033	-0.14 (-0.24, -0.04)	0.007	-0.11 (-0.21, -0.01)	0.044
Premature birth [b]	1.23 (0.97, 1.56)	0.091	1.07 (0.77, 1.49)	0.693	0.86 (0.60, 1.23)	0.407
SGA and LGA						
SGA [b]	0.86 (0.66, 1.13)	0.282	1.21 (0.87, 1.69)	0.263	1.23 (0.88, 1.72)	0.218
LGA [b]	1.52 (1.31, 1.78)	<0.001	1.09 (0.86, 1.38)	0.477	1.36 (1.08, 1.72)	0.009

[a] Adjusted for: maternal age, education, household income, BMI before pregnancy, gestational weight gain, first pregnancy, complications from previous pregnancy, insulin injection and gestational week; [b] Adjusted for: maternal age, education, household income, BMI before pregnancy, gestational weight gain, first pregnancy, complications from previous pregnancy and insulin injection; [c] After evaluating Pearson Chi-Square and C statistics, multivariate regression models showed an appropriate goodness of fit.

Supplementary table S4. Association of blood glucose levels and maternal weight before delivery (N=10010).

	Univariate analysis		Multivariate analysis[a]	
	β (95%*CI*)	*P*	Adjusted *β* (*95%CI*)	*P*
FPG	3.51 (2.24, 4.77)	<0.001	1.83 (0.56, 3.10)	0.005
1-h PG	0.55 (0.22, 0.88)	0.001	0.42 (0.09, 0.75)	0.012
2-h PG	-0.27 (-0.70, 0.17)	0.235	-0.28 (-0.72, 1.63)	0.202

[a] Adjusted for: maternal age, education, household income, BMI before pregnancy, gestational weight gain, first pregnancy, complications from previous pregnancy, insulin injection and gestational week.

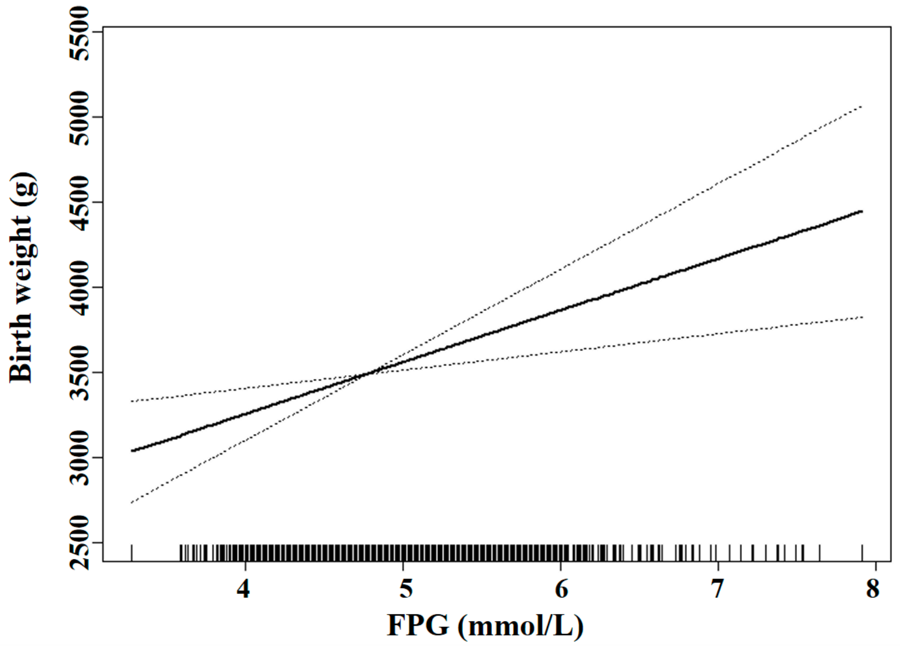

Supplementary figure S1. Association of FPG with birth weight by smoothing spline. Adjusted for: squared FPG.
